# Supplementary material for: Control of non-REM sleep by ventrolateral medulla glutamatergic neurons projecting to the preoptic area
Source: Nat Commun. 2022 Aug 12;13:4748. doi: 10.1038/s41467-022-32461-3 (PMC9374761; doi:10.1038/s41467-022-32461-3)
Supplement: Supplementary file 1 — Supplementary Information [file 41467_2022_32461_MOESM1_ESM.pdf]

SUPPLEMENTAL INFORMATION

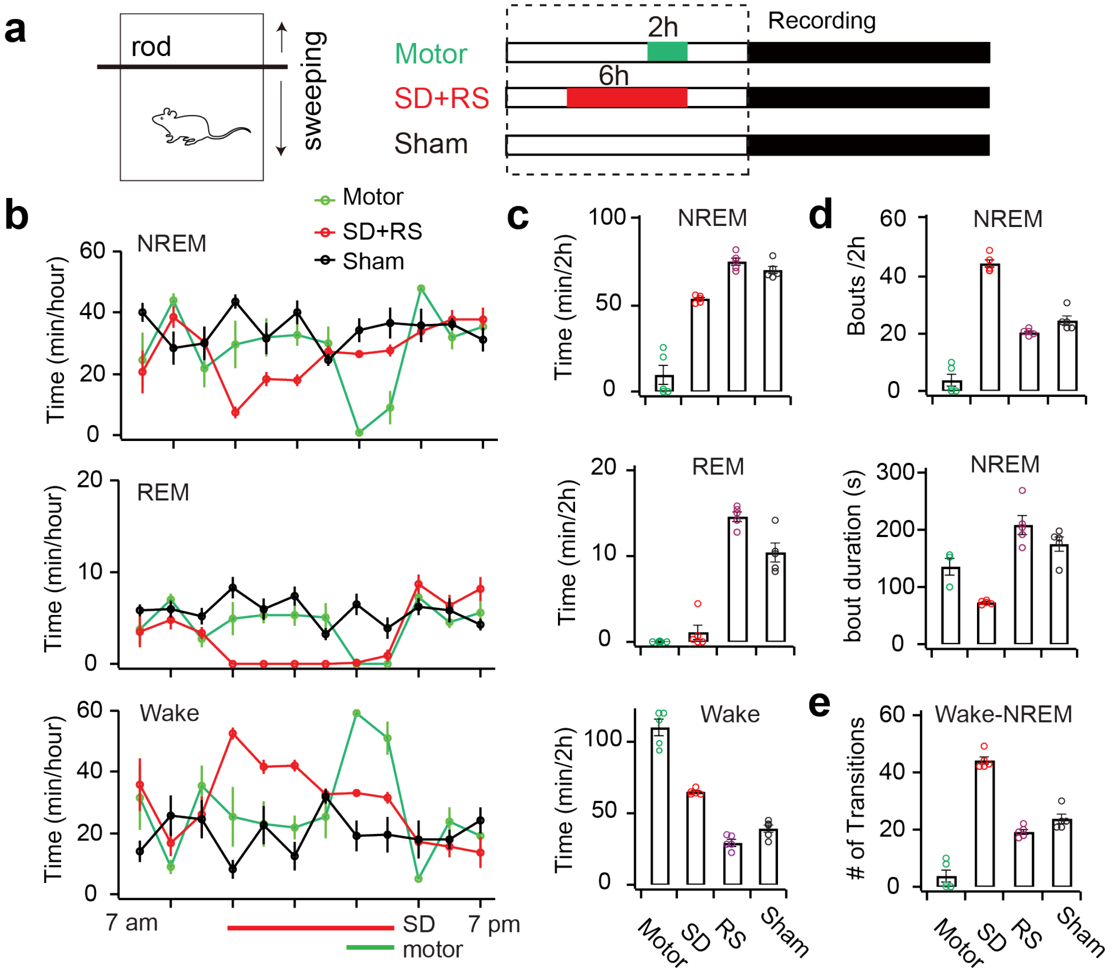

Supplementary Fig 1. Sleep patterns in sleep-deprived mice. a, Schematic of experimental design. Mice were divided into three groups and subject to different treatments: 2h motor control, 6h sleep deprivation (SD) followed by 2h recovery sleep (RS), and Sham control. Individual mice were placed in a behavioral chamber and recorded with EEG and EMG in the light cycle. **b**, Hourly sleep pattens in different groups before, during, and after treatments (indicated by colored lines for each group). Data are mean ± SEM (n = 5 mice for each group). **c**, Quantification of the total duration of NREM sleep, REM sleep, and wake in the 2-h window before the end of treatments. RS data were calculated from the 2-h recovery sleep in SD mice. **d**, Quantification of NREM sleep bouts and bout durations in the last 2-h window of treatments. **e**, Quantification of transitions from wake to NREM sleep in each group. Note the high transitions in the SD group. (n = 5 mice for each group). Data are mean ± SEM in panel **c, d, e**. Source data are provided as a Source Data file.

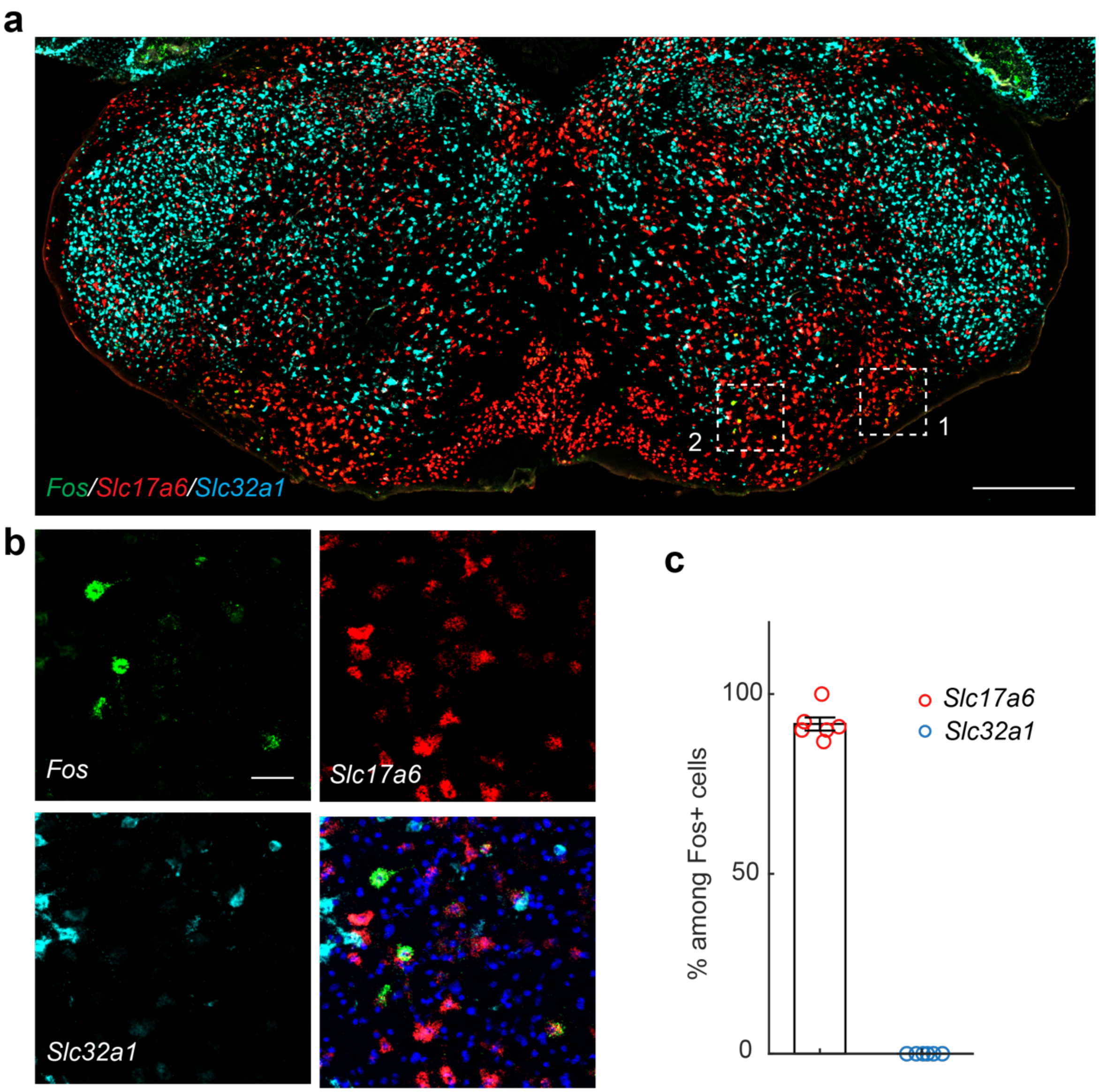

Supplementary Fig 2. Fluorescence *in situ* hybridization (FISH) in the medulla. a, A representative example of FISH of *Fos* and neuronal markers in a brain section of a SD mouse (representative of n = 2 mice). Box1 indicates the area shown in the **Fig.1d**. Box2 indicates the area enlarged in panel **b**. *Slc17a6*, vesicular glutamate transporter 2; *Slc32a1*, vesicular GABA transporter. Blue, DAPI. Scale bars, 500 µm in a and 50 µm in b. **c**, Quantitation of co-staining of *Slc17a6* and *Slc32a1* with *Fos* (6 brain sections in 2 mice). Error bars, SEM. Source data are provided as a Source Data file.

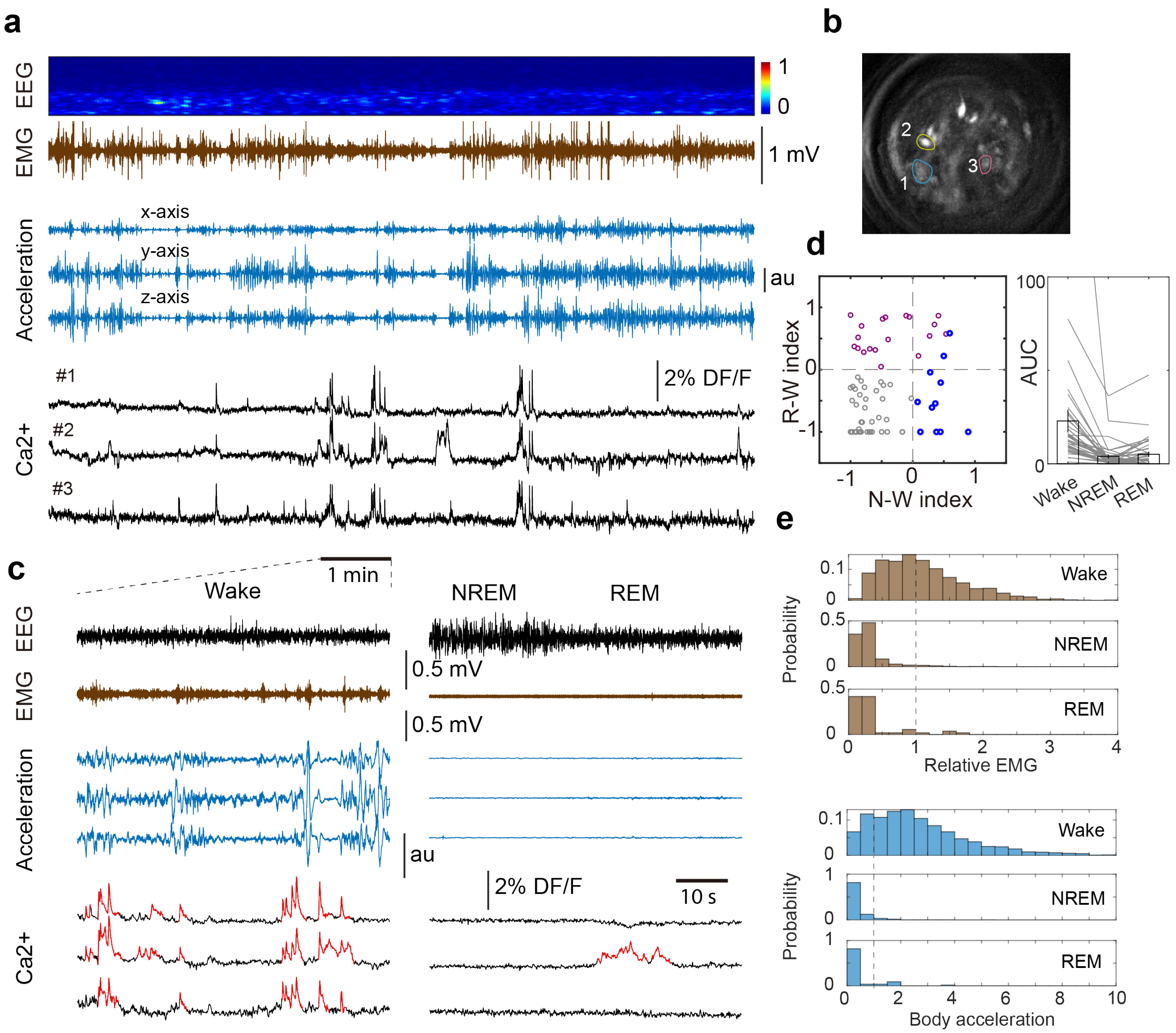

Supplementary Fig 3. Characterization of wake-related glutamatergic cells in the VLM. a, A representative example showing EEG power spectrogram (0-25Hz), EMG, body accelerations, and calcium traces in a wake imaging session. **b,** Activity map in a field of view indicates the location of 3 cells shown in a. **c**, Enlarged windows showing EEG, EMG, accelerations, and calcium activity in 3 motor-related cells during wake (left) and sleep (right) states. Detected calcium events are highlighted in red. **d**, Left, NREM-Wake (N-W) selectivity index versus REM-Wake (R-W) selectivity index in all 70 identified cells from 4 mice. Each symbol represents one neuron. Blue, NREM sleep active neurons (n = 11 neurons). Purple, REM sleep active neurons (n = 19). Gray, wake active neurons (n = 40). Right, Quantification of calcium activity of 40 wake-active cells (from 4 Vglut2-Cre mice) in different brain states. Activity was presented as area under curve (AUC). **e**, Distribution of calcium events in different levels of

EMG amplitude (upper) and body accelerations (lower) in each brain state. EMG was normalized to the average during wake in each mouse (i.e., 1 indicates the average EMG amplitude). Boy acceleration was first calculated from x, y, z-axis and then normalized to the average acceleration during wake. See methods for details. Source data are provided as a Source Data file.

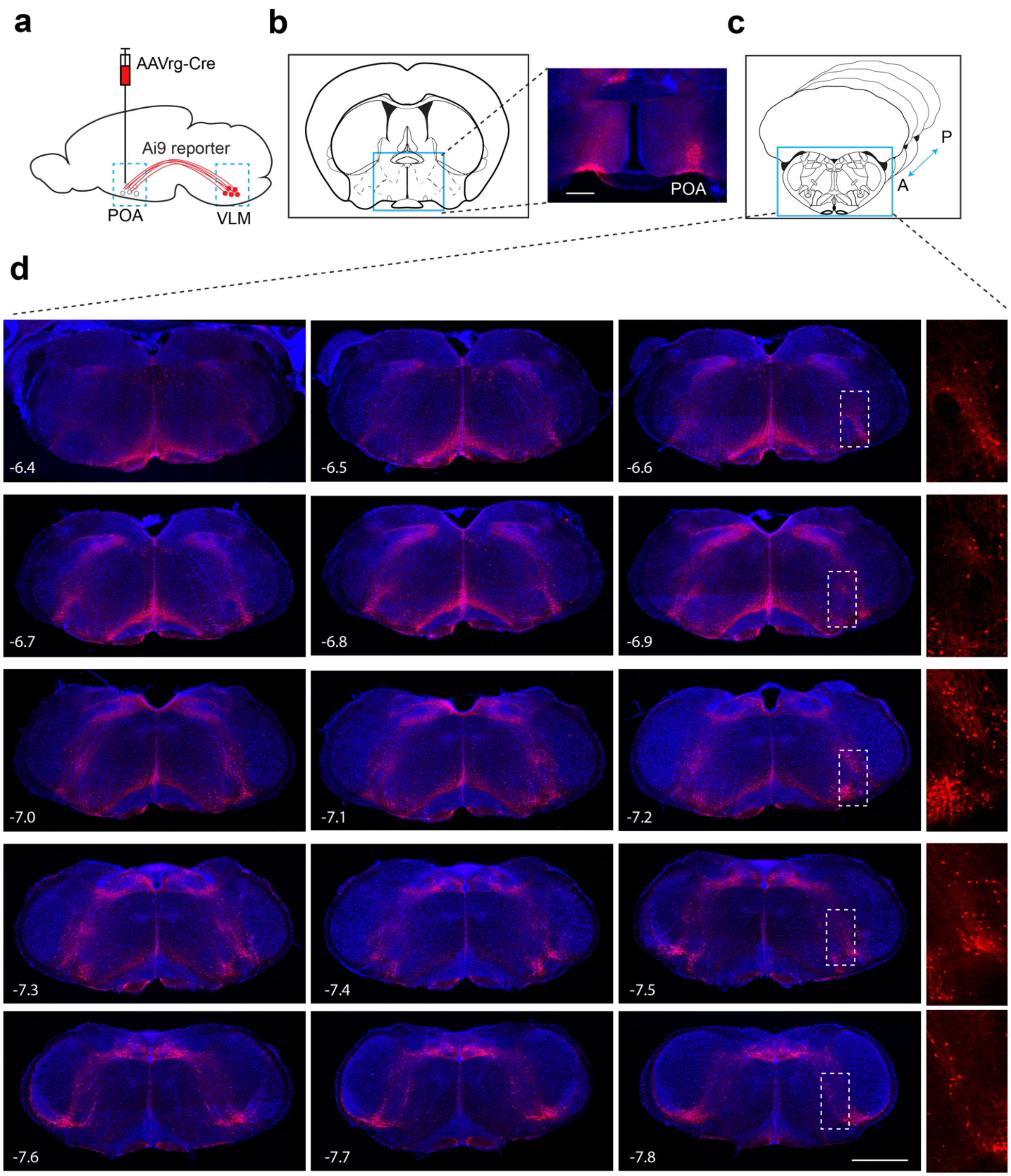

Supplementary Fig. 4 POA-projecting neurons in the brainstem. a, Schematic of retrograde labelling in Ai9 reporter mice injected with AAVrg-hSyn.Cre.WPRE.hGH into the preoptic area (POA). VLM, ventrolateral medulla. **b**, Fluorescence image illustrating bilateral viral injection sites in the POA (3 mice in total). Blue, DAPI. Red, tdTomato. **c-d**, Serial coronal images acquired by confocal microscopy showing the anatomy of retrogradely labelled neurons in the

brainstem (anterior to posterior: Bregma -6.4mm to -7.8mm, 3 mice in total). Regions with dashed boxes enlarged on the right. Mouse brain figures in **b** and **c** are adapted from Allen mouse brain atlas (http://atlas.brain-map.org). Scale bar, 1 mm.

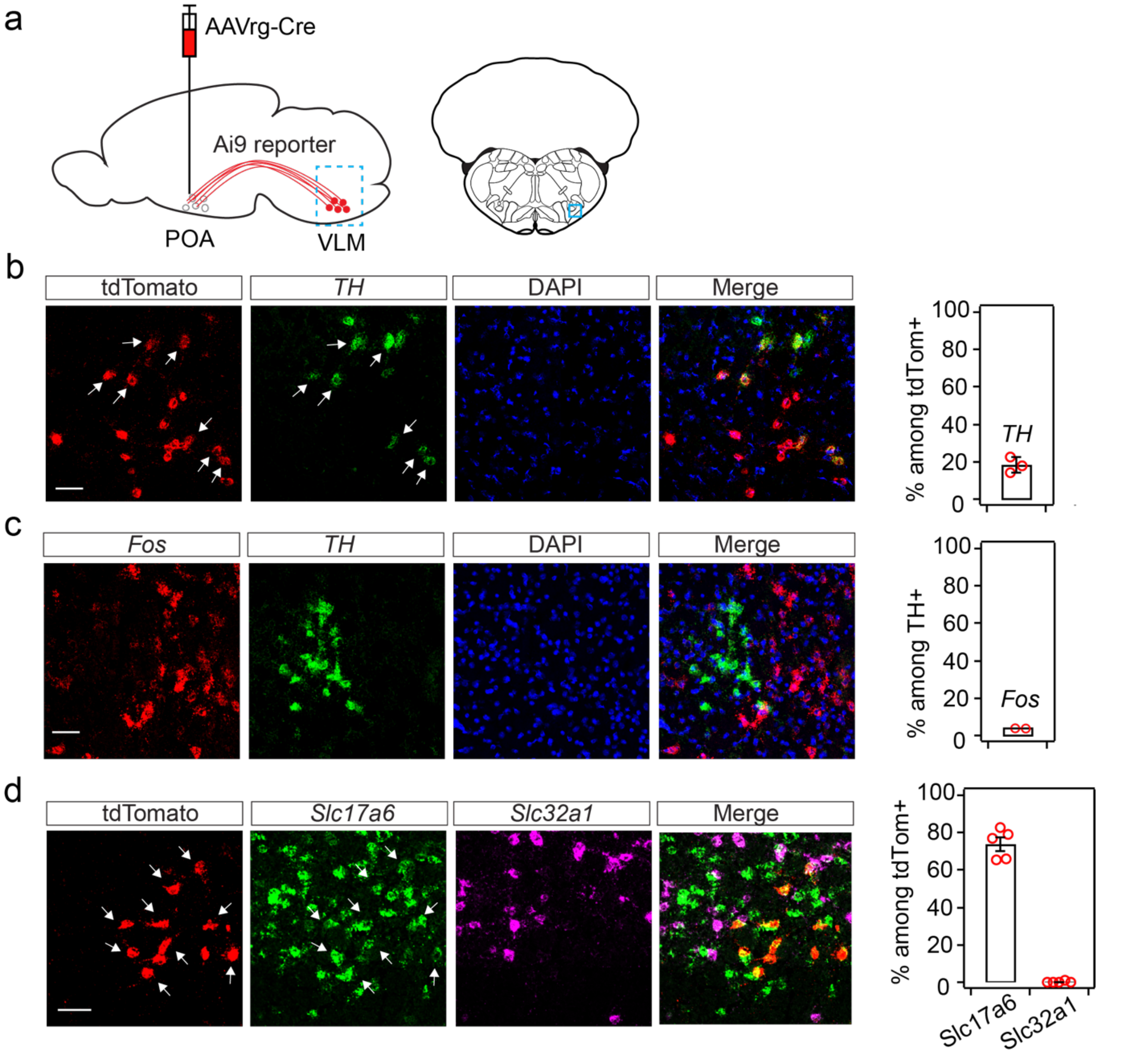

Supplementary Fig. 5 Medulla glutamatergic neurons, but not GABAergic neurons project to the POA. a, Schematic of retrograde tracing in Ai9 mice injected with AAVrg-Cre in the POA. Right, coronal section (Bregma -6.8 mm, adapted from Allen mouse brain atlas) showing the VLM region in panel **b, c, d**. **b**, Double fluorescence in situ hybridization (FISH) of tdTomato and tyrosine hydroxylase (TH) in the VLM. Arrows indicated the overlapped cells

between tdTomato and TH. Scale bars, 50 µm. Right, Quantitation of percentages of TH+ cells among retrogradely labeled cells (n = 3 animals). **c**, Double FISH of Fos (red) and TH (green) in the VLM. Scale bars, 50 µm. Right, Quantitation of percentages of *Fos*+ cells among TH+ cells (n = 2 animals). **d**, Triple FISH of tdTomato (retrograde labeling) and neuronal markers in the VLM. *Slc17a6*, vesicular glutamate transporter 2; *Slc32a1*, vesicular GABA transporter. Arrows indicated the overlapped cells between tdTomato and *Slc17a6*. Scale bars, 50 µm. Right, Quantitation of percentages of cell types among retrogradely labeled cells (n = 5 animals). Data are mean ± SEM in panel **b, c, d**. Source data are provided as a Source Data file.

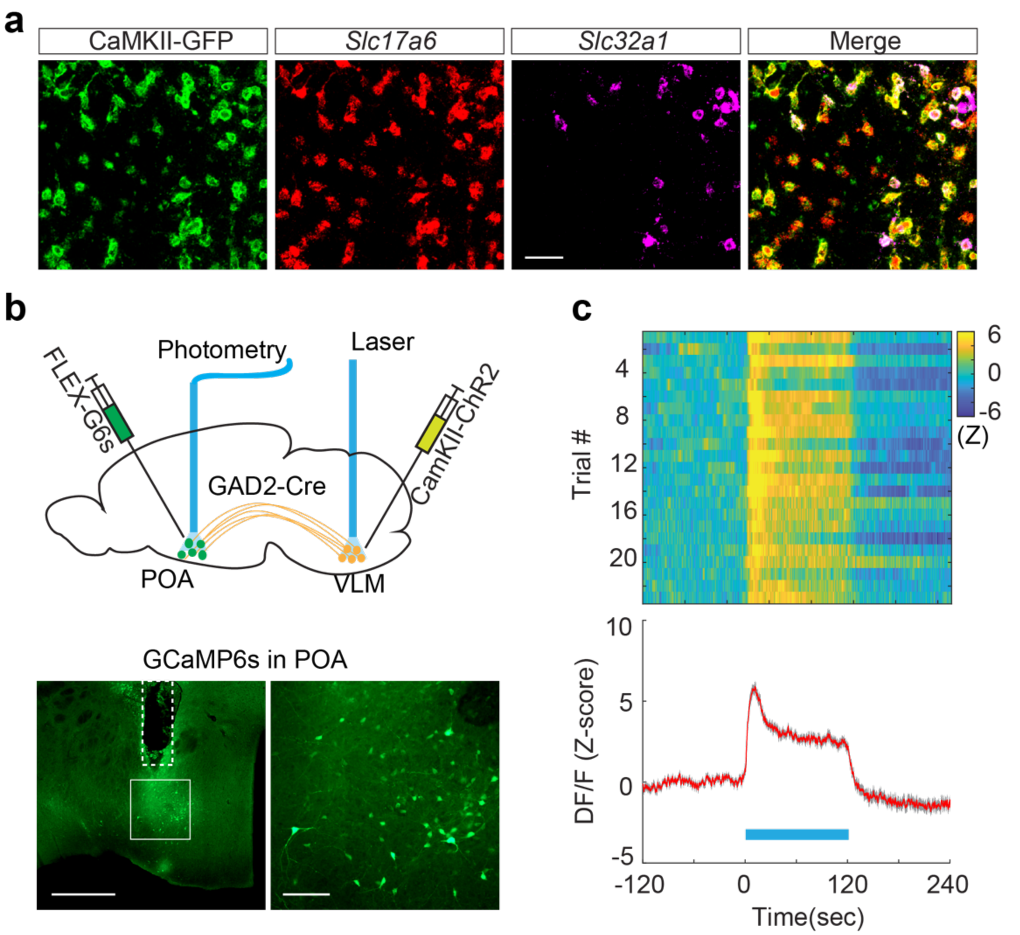

Supplementary Fig. 6 Medulla neurons activate POA GABAergic neurons. **a**, A representative example of fluorescence in situ hybridization (FISH) of CaMKII-GFP and neuronal markers in the VLM of a mouse injected with AAV9-CaMKII-eGFP (total 3 mice, 79.67% ± 2.00% overlap between CaMKII-GFP with *Slc17a6*, 12.10 ± 0.76% overlap with *Slc32a1*). *Slc17a6*, vesicular glutamate transporter 2; *Slc32a1*, vesicular GABA transporter. Scale bars, 50 µm. **b**, Schematic of experimental design. AAV9-CamKII-ChR2 was injected into the VLM, AAV1-FLEX-GCaMP6s was injected into the POA of GAD2-Cre mice. Bottom, GCaMP6s expression in the POA. Dashed box indicates the placement of an optic fiber. The region underneath of the optic fiber (solid-line box) is enlarged on the right. Scale bars, 0.5 mm and 0.1 mm, respectively. **c**, upper, Raster plots showing photometric signals of POA GABAergic neurons activated by optogenetic activation of medulla neurons. Lower, averaged (red) traces of photometric signals. Shadow, SEM. Blue bar marks light stimulation (20 Hz, 2 min). Photometric data were obtained from 23 trials in 4 GAD2-Cre animals and normalized to Z scores.

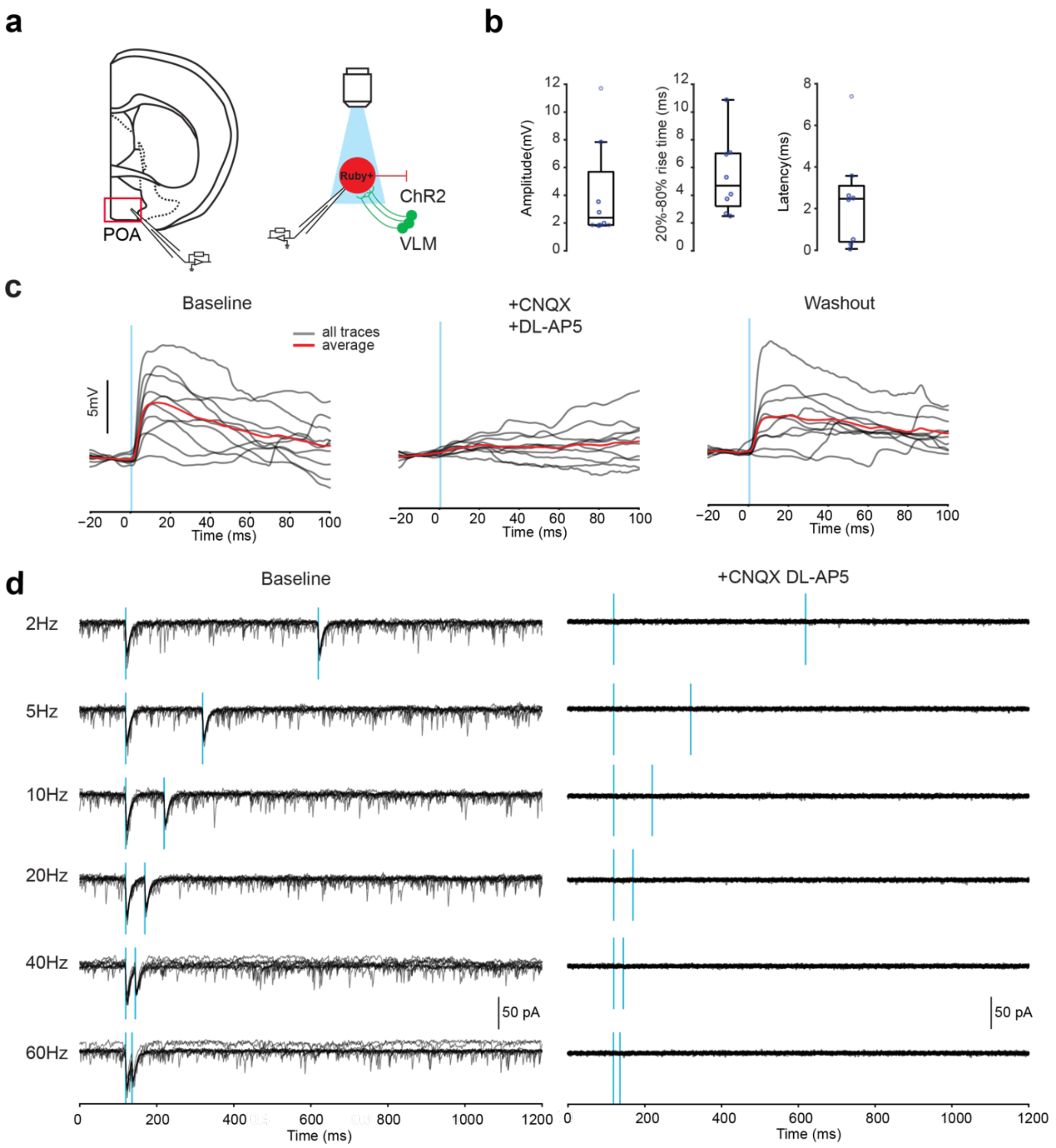

Supplementary Fig. 7 Slice recording in POA GABAergic neurons. a, Schematic of experimental design. Recordings were performed in mRuby+ cells in the POA while light stimulation was delivered to activate ChR2-expressing VLM glutamatergic axons. **b**, Quantitation of EPSP amplitudes, rise time, and latency in baseline recording shown in **Fig 4c** (n = 8 cells from 6 Vglut2-Cre mice). On each box, the central mark indicates the median, the bottom and top edges indicate the 25th and 75th percentile, the whiskers extend to the most extreme datapoints not considered outliers. **c**, A representative cell showing optogenetic-evoked EPSPs before (baseline) and after glutamatergic receptor antagonists CNQX (10 µM) and DL-AP5 (10 µM). Red, average; gray, individual sweeps. **d**, Example traces (10 sweeps in each

condition) of excitatory post-synaptic currents (EPSCs) with and without CNQX (10 μM) and DL-AP5 (10 μM). Blue lines indicate the light stimulation (1 ms). Source data are provided as a Source Data file.

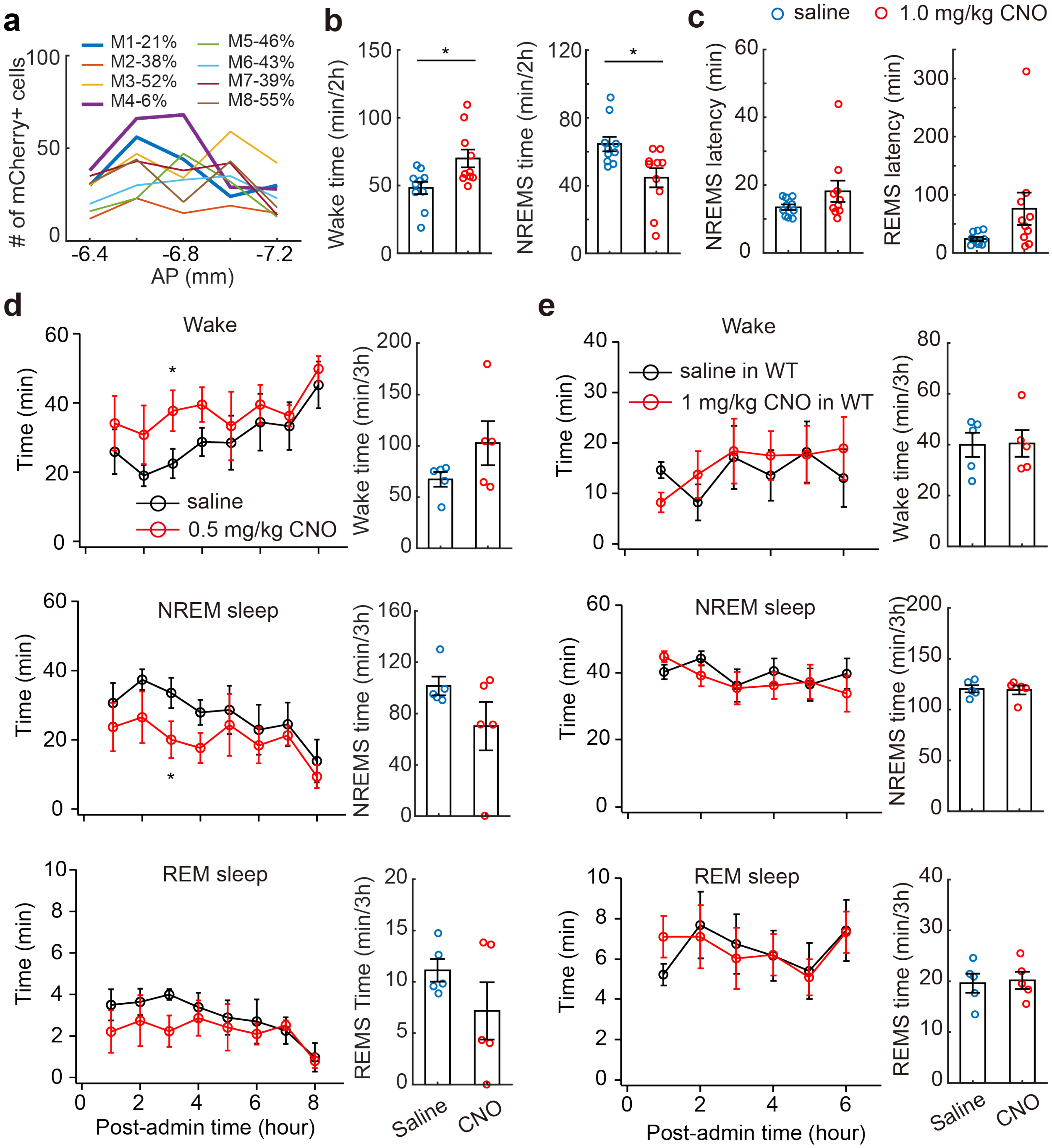

Supplementary Fig. 8 Chemogenetic inhibition of VLM neurons. a, Quantification of transduced cells (mCherry+) in chemogenetic inhibition (8 out of 10 mice shown in **Fig. 4**, no histology data for the rest 2 mice). Shown above are mouse ID and the percent of NREM sleep time over the 3-h post-CNO period. **b**, Quantitation of wake and NREM sleep time over the 2-h window after 1mg/kg CNO and saline treatments (n=10 mice, *, P=0.043 for wake time, P=0.040 for NREM time). **c**, Quantitation of NREM and REM sleep latency in mice treated with

1mg/kg CNO and saline (n=10 mice). **d**, Effects of CNO (0.5 mg/kg, i.p., red circles) and saline (black circles) treatment on the total wake, NREM sleep, and REM sleep time in mice injected with hM4Di (n = 6 mice, * P=0.033 for Wake, P=0.042 for NREM). **e**, Effects of CNO (1 mg/kg, i.p., red circles) and saline (black circles) treatment on the total wake, NREM sleep, and REM sleep time (n = 5 mice) in wild type mice (non-DREADDs). (n = 5 animals, no significance). Error bars, SEM. Two-sided paired t-test are used for comparisons in **b-e**. Source data are provided as a Source Data file.

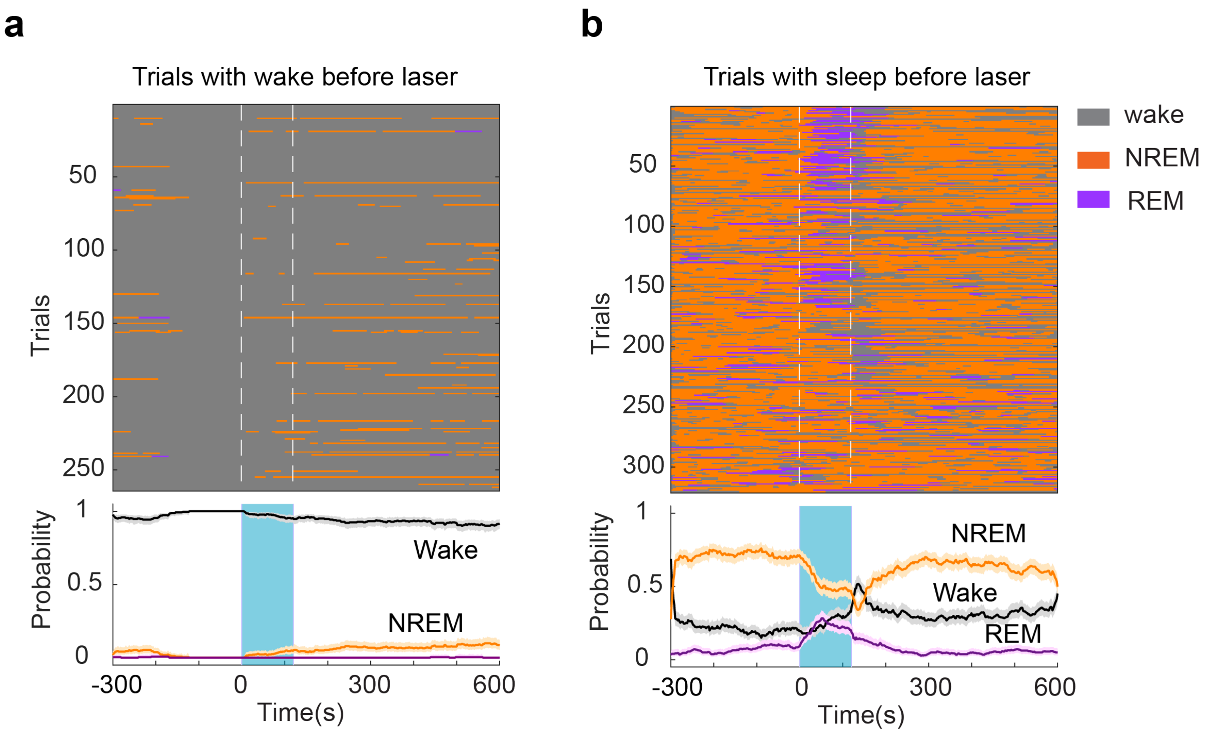

Supplementary Fig. 9 Optogenetic activation of VLM GABAergic neurons. **a**, Brain states (upper) and probability (bottom) of wake, NREM sleep, and REM sleep before, during and after laser stimulation (20 Hz, 2 min) in trials with wakefulness before laser (n = 6 GAD2-Cre mice). Bottom, probability of wake (black), NREM sleep (orange), and REM sleep (purple). Error bands, 95% bootstrap confidence intervals. **b**, Brain states (upper) and probability (bottom) of wake, NREM sleep, and REM sleep before, during and after laser stimulation in trials with sleep before laser stimulation (n = 6 GAD2-Cre mice). Error bands, 95% bootstrap confidence intervals. Note that optogenetic activation of VLM GABAergic neurons induced REM sleep in parts of trials.

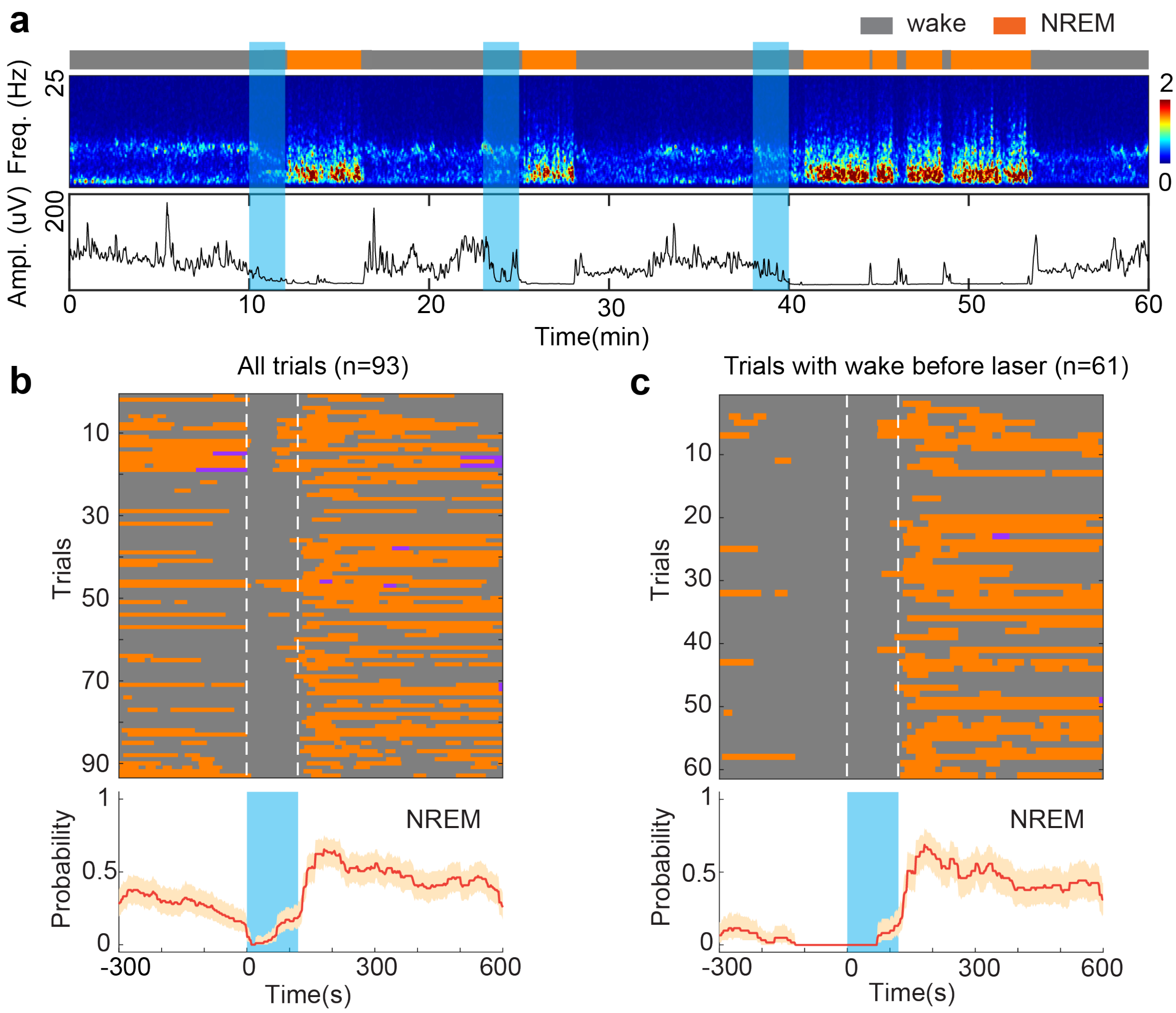

Supplementary Fig. 10 Optogenetic activation of CaMKII-labeled medulla neurons induced the transitions from wakefulness to NREM sleep. a. examples of optogenetic activation in a wild type mouse (C57BL/6J) injected with AAV9-CaMKII-ChR2. Upper panel, gray for wake, orange for NREM sleep. Middle panel, EEG power spectrogram (0-25 Hz). Lower panel, EMG amplitude. Blue strips in the lower panel indicate laser stimulation (2 min, 20 Hz). **b**, brain states in all trials (n=93) from five mice injected with AAV. The purple indicates REM sleep. Dashed white lines indicate the period of laser stimulation (2 min). The lower panel is the probability of NREM sleep before, during and after laser stimulation. **c**, similar to **b**, brain states in trials (n=61) when animals were awake during the 2 min before the onset of laser stimulation. Traces in lower panels of **b** and **c** are presented as mean ± 95% bootstrap confidence intervals (shading).

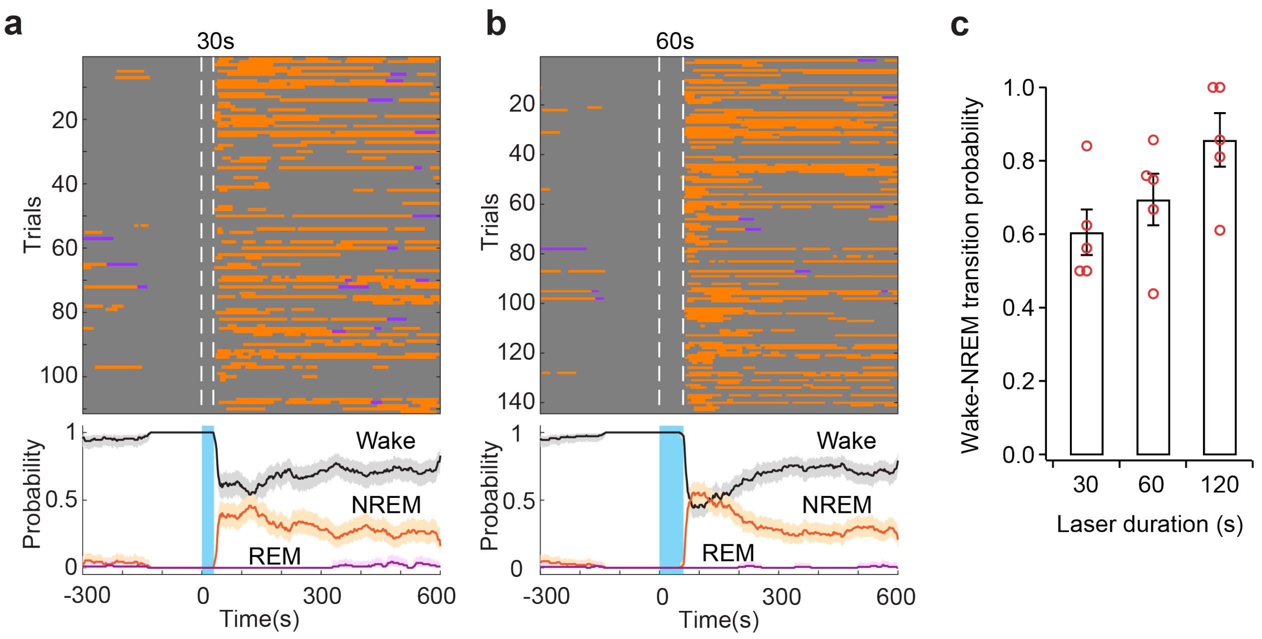

Supplementary Fig. 11 Optogenetic stimulation of VLM glutamatergic neurons. a, b, Brain states (upper) and probability (bottom) of wake (gray), NREM sleep (orange), and REM sleep (purple) in trials with wakefulness before laser stimulation (30 s and 60 s, 20 Hz) from 5 Vglut2-Cre animals injected with AAV1-DIO-ChR2-eYFP in the VLM. Traces in lower panels are presented as mean ± 95% bootstrap confidence intervals. **c**, Quantitation of wake-sleep transition probability with different durations of laser stimulation (n = 5 mice). Error bars, SEM. Source data are provided as a Source Data file.

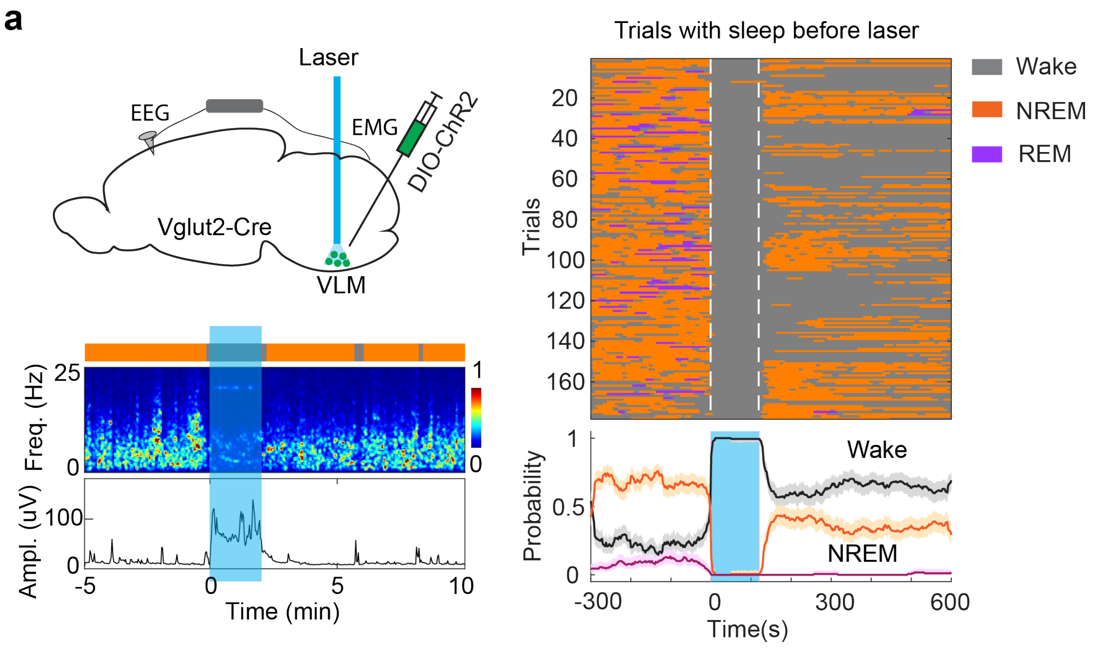

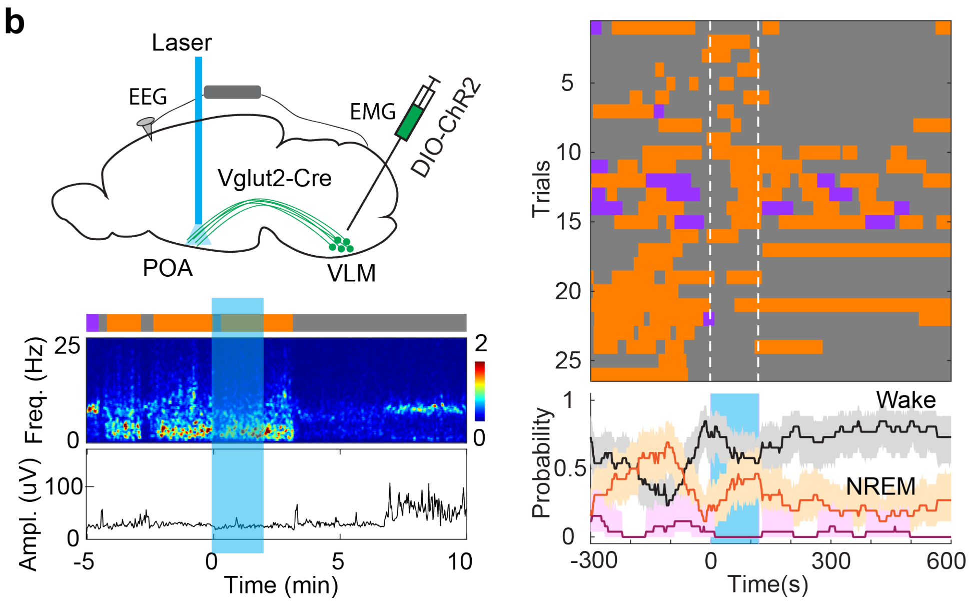

Supplementary Fig. 12. Optogenetic activation in sleeping mice. a, Optogenetic activation of VLM glutamatergic neurons interrupted sleep in sleeping animals. Left upper, schematic of experimental design. Left bottom, a trial of optogenetic activation (20 Hz, 2 min) in a sleeping mouse. Right, brain states (upper) and probability (bottom) before, during, and after light stimulation (20Hz, 2min) in trials with sleep before laser stimulation (n = 14 Vglut2-Cre animals). Traces are presented as mean ± 95% bootstrap confidence intervals (shading). **b**, Optogenetic activation of VLM glutamatergic terminals in the POA. Left, schematic of

experimental design and a trial of optogenetic activation. Right, brain states (upper) and probability (bottom, mean ± 95% bootstrap confidence intervals) of brain states before, during, and after light stimulation (20 Hz, 2 min) in trials with sleep before laser stimulation (n = 7 Vglut2-Cre animals).

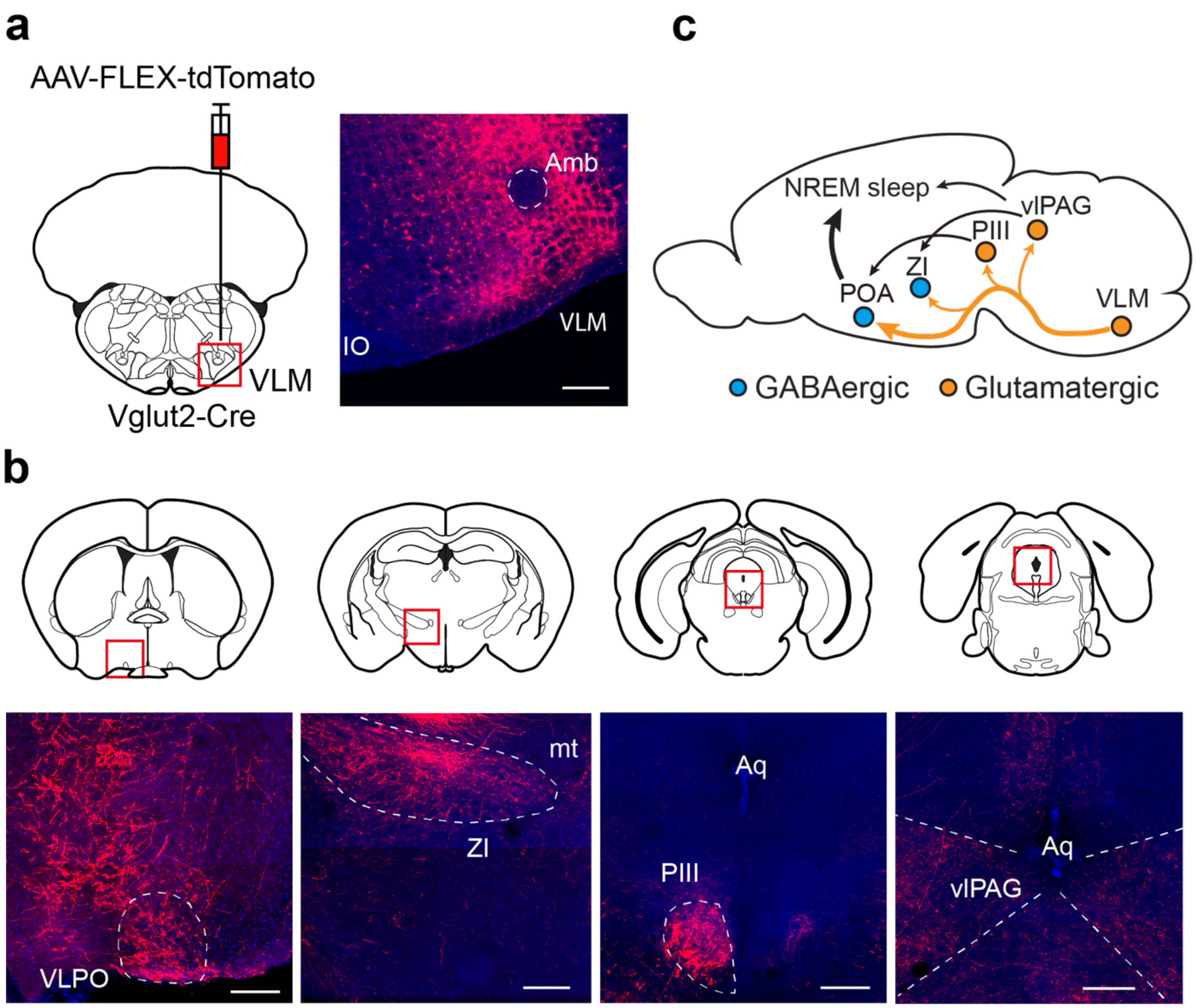

Supplementary Fig. 13. Anterograde tracing from VLM glutamatergic neurons. a, Schematic of anterograde tracing experiment. AAV1-CAG-FLEX-tdTomato was unilaterally injected in the VLM of Vglut2-Cre mice. Right, expression of tdTomato at the injection site in the VLM of a Vglut2-Cre mouse (n=3 mice). **b**, Representative VLM glutamatergic terminals in the POA, ZI, PIII, vlPAG (total 3 Vglut2-Cre mice). Mouse brain figures adapted from Allen mouse brain atlas (http://atlas.brain-map.org). Amb, nucleus ambiguus; IO, inferior olivary complex; Aq, aqueduct; mt, mammillothalamic tract; ZI, zona incerta, vlPAG, ventrolateral periaqueductal gray. Scale bars, 200 μm. **c**, A model of the VLM network that promotes NREM sleep. The orange lines show the excitatory projections from the VLM to other NREM-promoting brain regions. Connections among downstream regions (black lines) are adapted from previous studies. NREM-related brain regions not directly connected to the VLM are not included.

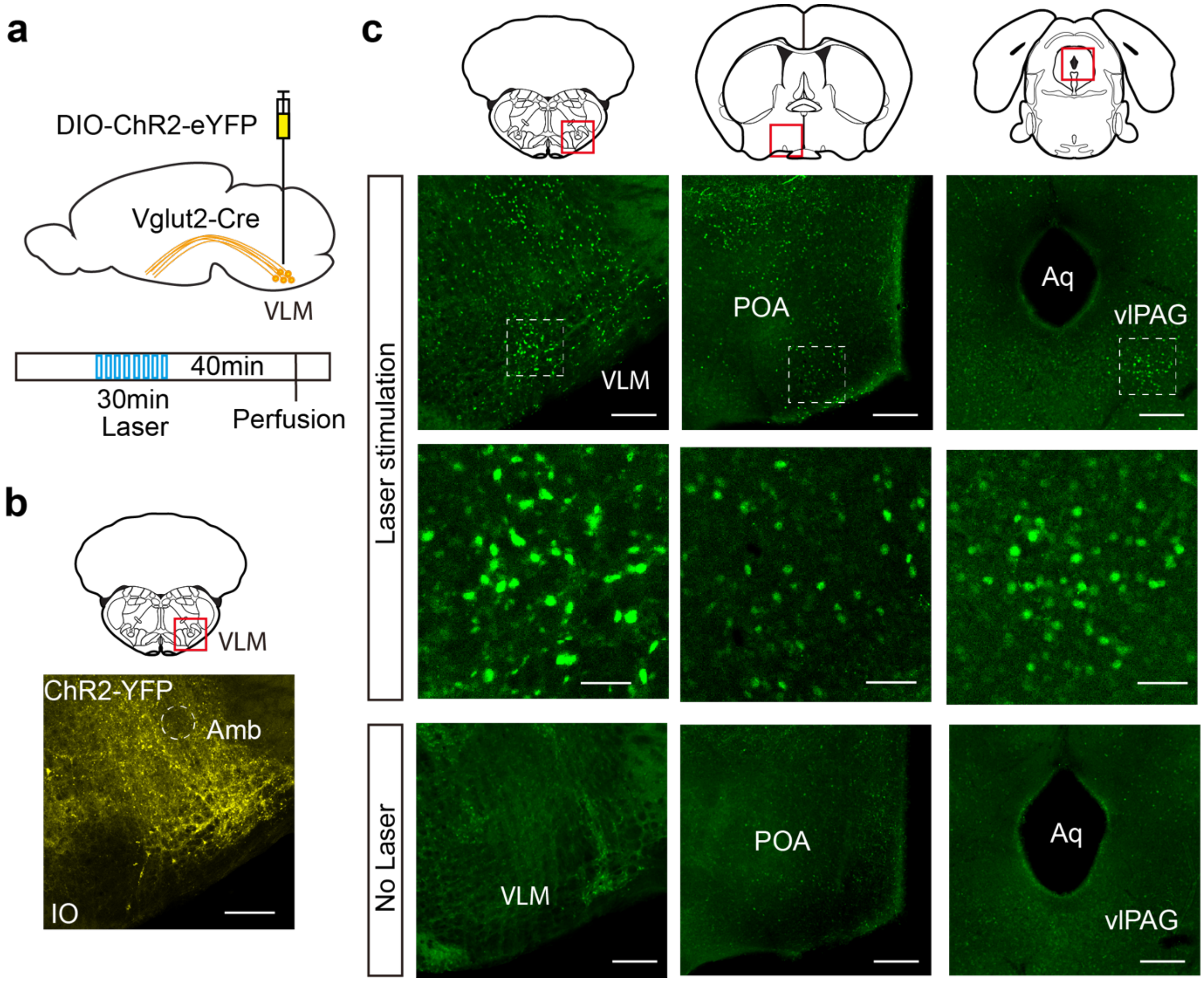

Supplementary Fig. 14. Optogenetic-induced cFos activity in the downstream targets of VLM glutamatergic neurons. a, Schematic of experimental design. Laser stimulation (20 Hz, 30 min in total) was delivered to the VLM neurons in Vglut2-Cre mice unilaterally injected with AAV-DIO-ChR2-eYFP in the VLM. Mice were perfused 40 min after the offset of laser stimulation. **b**, Expression of ChR2-YFP at the injection site in the VLM (cFos expression in the same area is shown in panel **c**, 2 mice in total). Scale bar, 200 µm. **c**, Representative fluorescence images of c-Fos immunostaining in the VLM, POA, and vlPAG in a ChR2 mouse (upper) and a control mouse (total 2 mice for laser stimulation, 2 mice for no laser control). Mouse brain figures adapted from Allen mouse brain atlas. Amb, nucleus ambiguus; IO, inferior olivary complex; Aq, aqueduct; Scale bars, 200 µm (upper and bottom panels), 50 µm (middle panel).

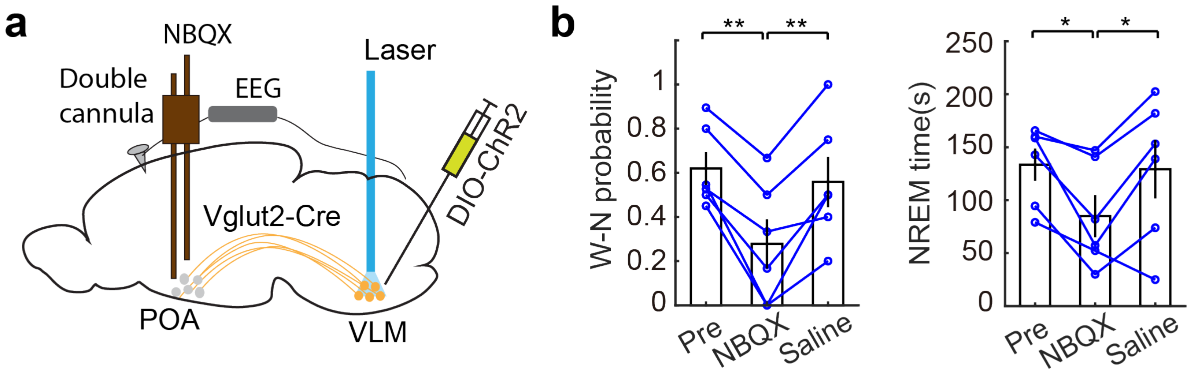

Supplementary Fig. 15. Pharmacological inhibition of POA neurons suppressed optogenetic-induced wake-sleep transitions. a, Schematic of experimental design. Vglut2-Cre mice were injected with AAV-DIO-ChR2-eYFP and implanted with an optic fiber unilaterally in the VLM, then implanted with a double cannula bilaterally above the POA. **b**, Left, Quantitation of optogenetic-induced wake (W) to NREM (N) transition probability before (pre) and after NBQX/saline injection in the POA (6 sessions from 4 animals). Each line represents one test session. Right, Quantitation of optogenetic-induced NREM sleep before (pre) and after NBQX/saline treatments. The amount of NREM sleep within a 5-min period following the offset of laser stimulation was calculated for comparison. Error bars, SEM. (** P=0.0016 between Pre and NBQX, P=0.0053 between NBQX and Saline in the left panel; * P=0.0158 between Pre and NBQX, P=0.0369 between NBQX and Saline in the right panel, two-sided paired t-test). Source data are provided as a Source Data file.
