# Supplementary material for: Control of non-REM sleep by ventrolateral medulla glutamatergic neurons projecting to the preoptic area
Source: Nat Commun. 2022 Aug 12;13:4748. doi: 10.1038/s41467-022-32461-3 (PMC9374761; doi:10.1038/s41467-022-32461-3)
Supplement: Supplementary file 4 — Reporting Summary [file 41467_2022_32461_MOESM4_ESM.pdf]

# nature portfolio

Corresponding author(s): Yueqing Peng

Last updated by author(s): Jul 11, 2022

# Reporting Summary

Nature Portfolio wishes to improve the reproducibility of the work that we publish. This form provides structure for consistency and transparency in reporting. For further information on Nature Portfolio policies, see our Editorial Policies and the Editorial Policy Checklist.

## Statistics

For all statistical analyses, confirm that the following items are present in the figure legend, table legend, main text, or Methods section.

| n/a | Confirmed | |
|---|---|---|
| ☐ | ☒ | The exact sample size (*n*) for each experimental group/condition, given as a discrete number and unit of measurement |
| ☐ | ☒ | A statement on whether measurements were taken from distinct samples or whether the same sample was measured repeatedly |
| ☐ | ☒ | The statistical test(s) used AND whether they are one- or two-sided<br>*Only common tests should be described solely by name; describe more complex techniques in the Methods section.* |
| ☒ | ☐ | A description of all covariates tested |
| ☐ | ☒ | A description of any assumptions or corrections, such as tests of normality and adjustment for multiple comparisons |
| ☐ | ☒ | A full description of the statistical parameters including central tendency (e.g. means) or other basic estimates (e.g. regression coefficient) AND variation (e.g. standard deviation) or associated estimates of uncertainty (e.g. confidence intervals) |
| ☐ | ☒ | For null hypothesis testing, the test statistic (e.g. *F*, *t*, *r*) with confidence intervals, effect sizes, degrees of freedom and *P* value noted<br>*Give P values as exact values whenever suitable.* |
| ☒ | ☐ | For Bayesian analysis, information on the choice of priors and Markov chain Monte Carlo settings |
| ☒ | ☐ | For hierarchical and complex designs, identification of the appropriate level for tests and full reporting of outcomes |
| ☒ | ☐ | Estimates of effect sizes (e.g. Cohen's *d*, Pearson's *r*), indicating how they were calculated |

*Our web collection on statistics for biologists contains articles on many of the points above.*

## Software and code

Policy information about availability of computer code

| | |
|---|---|
| Data collection | All commercial software to collect the data in this study are described, including the Neuralynx and TDT systems for sleep recording, the TDT system for fiber photometry, the Inscopix software for microendoscopic calcium imaging, pClamp for slice recording, and Zeiss confocal for imaging. |
| Data analysis | The IDPS (Inscopix) and ImageJ were used for imaging processing. Custom-built MATLAB codes were used for various analyses. We described key parameters in the method section. |

For manuscripts utilizing custom algorithms or software that are central to the research but not yet described in published literature, software must be made available to editors and reviewers. We strongly encourage code deposition in a community repository (e.g. GitHub). See the Nature Portfolio guidelines for submitting code & software for further information.

## Data

Policy information about availability of data

All manuscripts must include a data availability statement. This statement should provide the following information, where applicable:

- Accession codes, unique identifiers, or web links for publicly available datasets
- A description of any restrictions on data availability
- For clinical datasets or third party data, please ensure that the statement adheres to our policy

Source data are provided with this paper. All other data supporting the findings of this study are available from the corresponding author upon reasonable request.

## Human research participants

Policy information about studies involving human research participants and Sex and Gender in Research.

| | |
|---|---|
| Reporting on sex and gender | N/A |
| Population characteristics | N/A |
| Recruitment | N/A |
| Ethics oversight | N/A |

Note that full information on the approval of the study protocol must also be provided in the manuscript.

# Field-specific reporting

Please select the one below that is the best fit for your research. If you are not sure, read the appropriate sections before making your selection.

[x] Life sciences   [ ] Behavioural & social sciences   [ ] Ecological, evolutionary & environmental sciences

For a reference copy of the document with all sections, see nature.com/documents/nr-reporting-summary-flat.pdf

# Life sciences study design

All studies must disclose on these points even when the disclosure is negative.

| | |
|---|---|
| Sample size | Sample sizes were determined by our pilot experiments and literature. Sample sizes were provided in the main text or figure legends. |
| Data exclusions | No data were excluded from the analyses. |
| Replication | Experiments were replicated multiple times with independent mice. The information has been included in figure legends. |
| Randomization | Selection of animals for experiments was randomized across litters, ages, and sexes. For optogenetic experiments, the sequence of with-light or without-light sessions were randomized. For pharmacological experiments, the sequence of NBQX and saline was randomized. |
| Blinding | The experimenters were blinded to control and experimental groups if possible. In chemogenetic experiments, experimenters were blinded to mouse groups. In optogenetic experiments, laser stimulation was automated by a MATLAB program. The analysts were not blinded to groups due to the labeling in data. Two persons independently reviewed the results and no subjective bias was found. In NBQX experiments, experimenters were not blinded to drugs due to the fixed drug administration schedule. |

# Reporting for specific materials, systems and methods

We require information from authors about some types of materials, experimental systems and methods used in many studies. Here, indicate whether each material, system or method listed is relevant to your study. If you are not sure if a list item applies to your research, read the appropriate section before selecting a response.

### Materials & experimental systems

| n/a | Involved in the study | |
|---|---|---|
| [ ] | [x] | Antibodies |
| [x] | [ ] | Eukaryotic cell lines |
| [x] | [ ] | Palaeontology and archaeology |
| [ ] | [x] | Animals and other organisms |
| [x] | [ ] | Clinical data |
| [x] | [ ] | Dual use research of concern |

### Methods

| n/a | Involved in the study | |
|---|---|---|
| [x] | [ ] | ChIP-seq |
| [x] | [ ] | Flow cytometry |
| [x] | [ ] | MRI-based neuroimaging |

## Antibodies

| | |
|---|---|
| Antibodies used | Guinea pig c-Fos antibody (1:1000, Synaptic systems, cat#226308), Alexa-488 donkey anti-guinea pig (1:500, Jackson ImmunoResearch, cat#706-545-148) |

| | |
|---|---|
| Validation | All antibodies used in this study were commercially available. The c-Fos antibody has been validated for IHC, IHC-P, ICC,WB, iDISCO in mouse, rat, human. |

## Animals and other research organisms

Policy information about studies involving animals; ARRIVE guidelines recommended for reporting animal research, and Sex and Gender in Research

| | |
|---|---|
| Laboratory animals | C57BL/6J (JAX 000664), VGlut2-IRES-Cre (JAX 028863), Gad2-IRES-Cre (JAX 0101802), Ai9 (JAX 007909), male and female, 8-20 weeks old in all strains |
| Wild animals | The study does not involve wild animals. |
| Reporting on sex | Both male and female animals are used in this study. |
| Field-collected samples | The study does not involve samples collected from the field. |
| Ethics oversight | All procedures were carried out in accordance with the US National Institute of Health (NIH) guidelines for the care and use of laboratory animals, and approved by the Animal Care and Use Committees of Columbia University. |

Note that full information on the approval of the study protocol must also be provided in the manuscript.
